# What Are the Conditions That Justify Oöphorectomy?

**Published:** 1883-05

**Authors:** Clinton Cushing


### Selections.

WHAT ARE THE CONDITIONS THAT JUSTIFY OÖPHORECTOMY? By CLINTON CUSHING, M. D. (Read before the S. F. Obtrical Society.)

By the term oöphorectomy is meant the removal of the ovaries for disease other than that of ovarian tumors, which increase rapidly, and tend to destroy life on account of their size. Ten years seem a short time for the history of an operation; yet this period includes all that is known of an operation that is being performed now in nearly all parts of the civilized world.

The original operators—Hegar of Germany, Lawson Tait, of England, and Dr. Robert Battey, of America—are still living; and the history of this precedure is still so fresh, and is doubtless so familiar to you all, that its repetition is unnecessary.

Like all new and radical operations, however, it has met with opposition and criticism from many members of the profession, and its position as a legitimate procedure is as yet not fully admitted; but as the roll of successful cases steadily grows larger, the opponents are gradually giving way, and the prospects are good that it will soon be recognized as the best if not the only means of curing a class of cases that have heretofore been looked upon as the opprobria of the profession.

The opposition to oöphorectomy is indeed slight as compared with the bitter war made upon the ovariatomists of forty years ago. At that time many leading professional men in Philadelphia called Dr. Washington Atlee a butcher, and little short of a murderer; and refused not only to be present at, or to countenance his operations for the removal of ovarian tumors, but refused to meet him in consultation in any case—but, as all know, he lived to see his name famous, and the operation endorsed even by his enemies.

A later writer says: "The history of ovariotomy ought to teach the surgery of the future humility, as its first lesson; and of the past, it must never be forgotten that whatever of defeat or obloquy it encountered, was due to it."

This older surgery was one of traditions. Its limits were defined by authority, its methods were settled by command, and had no place within its borders for whatever violated its dogmas. One of its most cherished dogmas was the sanctity of the peritoneum, and the inviolability of the great cavities. Whatever the older surgery taught about these things was tradition, not fact, and tradition it remained until the creation of a new science and art called ovariotomy. Nothing can be stronger than the contrast between the present attitude of general surgery toward ovariotomy, and that held twenty-five years ago. Whatever, at the present day, is proposed or attempted in abdominal and pelvic surgery is received with plaudits, and finds a score of hands to essay its difficulties. This influence is really not limited to abdominal surgery; its impetus is felt throughout the field, and many a triumph has been scored under the moral stimulus of ovariotomy.

When the size and location of the ovaries are considered, and it is remembered that their existence is not necessary, either to the life or health of the female, the influence which they exert, especially when diseased, upon the general economy is remarkable. Acting, as they do, upon distant parts of the body, through the medium of the sympathetic nerves, there is not an organ or a function that are not at times rendered morbid. Even the mind at times seems to be under the direct control of these small organs. If, then, the evil influence of diseased ovaries may be so great, their removal, as a means of curing disease that can be reached in no other way, comes to be a matter of greatest interest.

In considering the conditions that justify the operation, I should place first atresia of the vagina or uterus, where the canal cannot be kept open, and where the ovulation was attended by serious symptoms, such as excessive pain, and symptoms of shock, and hæmorrhage into the abdominal cavity.

It was upon a case of this kind that Dr. Battey performed the operation in 1878. The atresia was due to the extensive sloughing of the genital tract, consequent upon labor, the cicatricial contraction being so great that the canal could not be kept patent; or, in congenital absence of the vagina, when it is impractible to form a canal, or where the new formed canal cannot be made permanent, the removal of the ovaries may be necessary in order to restore health, or even to save life.

Uterine hæmorrhages to a degree involving danger to life or serious impairment of health, and incurable by the administration of medicine, or by non-operative procedure, are undoubtedly cases for oöphorectomy where the cause exists outside of the cavity of the uterus. Among the causes may be mentioned fibroid tumors of the uterus, the existence of small cysts of the ovaries, disease of the fallopian tubes, such as dropsy or accumulations of pus in their cavities, and the existence of chronic inflammation of the ovaries and the pelvic peritoneum or pelvic areolar tissue.

The success of Lawson Tait in the treatment of these cases is certainly remarkable. Of his last twenty-five cases there has been but one death, twenty ono were entirely cured, and three greatly improved. When these results are compared with other methods, such as hysterectomy, the use of electricity, the administration of ergot and bromide of potassium, and other means, it will be seen that no other method equals this in value. A peculiarity of Mr. Tait's method of operating is the removal with the ovaries of the fallopian tubes. He takes the ground that the monthly discharge of blood from the uterus is due, not to the influence of the ovaries, but to that of the fallopian tubes, and he bases his conclusions on the fact that in his cases where the ovaries alone were removed, the hæmorrhage from the uterus was not always controlled, but that when both the tubes and ovaries were removed, a cure followed. This is certainly a new departure from our previously accepted doctrines, and a further investigation by other observers will be required before Mr. Tait's views will be accepted.

A fact is adduced, and one that is well known to the profession, that ovulation is not necessarily attended by a discharge of blood from the uterus, for women frequently become pregnant while nursing, and before the reappearance of the menstruation after the delivery of their last child. Women have also been known to become pregnant before they have ever had a menstrual discharge.

Many other cases of the removal of the ovaries for the cure of metrorrhagia, due to the existence of fibroid tumors of the uterus, have been reported in the various medical journals during the past few years, and generally with good results, but none so favorably as the series reported by Mr. Tait.

Dr. T. Addis Emmet has recently visited Mr. Tait, in England, and he reports that from what he saw, he can speak favorably of Mr. Tait's work in this direction. The object of the operation in this class of cases is, that by the removal of the ovaries, we bring about the change of life, remove the influence of the ovaries and fallopian tubes on the nutrition of the uterus, lessen the blood supply to the parts and produce atrophy similar to that following the menopause. With the present light that we have on the subject, there can be no doubt that in properly-selected cases, the operation holds out a better prospect of cure than any other. The question of the removal of the uterine appendages for metrorrhagia due to chronic disease of the ovaries, or fallopian tubes, is still far from being settled, and it seems to me that it resolves itself first into the fact of our being able to diagnose, by means of an examination, whether there is sufficient disease of the appendages to warrant the belief that the fault lies with these organs. When the cysts in the ovaries are small, or the ovaries but little enlarged and not displaced, or when the fallopian tubes are not materially increased in size, many difficulties arise to prevent our gaining the desired knowledge. The abdominal walls may be rigid and unyielding, or thick and loaded with fat, or the pelvic structures may be more or less fixed and agglutinated by former inflammations, the distension of the abdomen by tympanites, or by ascitic fluid, all tend to perplex or hinder in the arrival at correct conclusions. In such an event, and we are unable to determine clearly the condition of the ovaries, if we can discover no other cause, and the hæmorrhage is serious enough to imperil life, or render the patient an invalid, then the removal of the ovaries is justifible if the loss of blood cannot be controlled by other means. Prolapsus of the ovary into Douglass' pouch, accompanied with enlargement, chronic inflammation and adhesion to the surrounding structures, is an affliction that is well nigh irremediable by any means except it be by its complete removal.

In a former paper read before this society I detailed the methods most likely to give relief in prolapse of the ovaries. If, however, we are unable to restore the patient to health by less severe means, and she was condemned to a life of invalidism by reason of her infirmity, the removal of the dislocated organ is justifiable.

In hernia of the ovary into or through the inguinal ring, when it is not reducible, and its abnormal position is attended by severe pain and swelling of the parts, and the heart becomes or is likely to become seriously impaired, the removal of the ovary is indicated.

All who have had much to do with the treatment of women have met with a considerable number of cases that have proved more than a match for their skill, and are classed under the general head of chronic pelvic inflammation. Commencing as a case of pelvic peritonitis or cellulitis, or pelvic hæmatocele, they end in partial recovery—perhaps in the formation of pelvic abscess. The recovery is never perfect, and from time to time, varying from weeks to months, there is a recurrence of the inflammation and pain until the health is destroyed, and the woman is left an invalid for the balance of her life. The treatment by general and local measures gives a modicum of relief, but a perfect cure is not arrived at; and the woman lives on from year to year a burden to herself and to all round her, with no hope of relief this side of the grave. In this class of cases Mr. Tait proposes the removal of the uterine appendages, in order to effect a cure, and he has now operated in thirty-five instances with but one death.

Regarding the secondary results, he says: "Precisely the same kind of argument applies to its secondary results, which in the hands of experienced operators are admittedly bad. For my own results, so far I have abundant cause for satisfaction. Some of my cases yet are incompletely relieved, but by far the majority of them are absolutely cured."

A noticeable feature of Mr. Tait's cures, is the fact that in nearly every one the fallopian tubes were diseased; they were either dropsical, or contained muco-purulent matter or pus, and that, too, when the ovaries were not markedly diseased, except that they were cirrhotic, apparently from the effect of previous inflammation. This new departure in the management of these cases is both bold and novel, and if it proves, after a fair trial, to cure a considerable proportion of those cases that have heretofore been looked upon as incurable, then may we encourage our patients to take the risk of the operation with the hope of a cure as the result.

If there is any one principle in surgery more clearly settled than another, it is that diseased and incurable organs, the existence of which is seriously impairing the health, shall be removed by the knife, so, after all, it is not so much the propriety of this operation as a justifiable surgical procedure in the case last spoken of that calls for discussion, but the point to be settled if possible, is the conditions when it is the best thing to do.

Under the heading of neuralgia, there is a group of painful affections that have their site in and about the pelvic organs of women. Ovarian neuralgia, neuralgic dysmenorrhœa, coccygodynia, vaginismus, intercostal neuralgia, especially under the left breast, pain in the top of the head, and reflex symptoms, generally of a neuralgic type.

It has been proposed in severe cases of neuralgic or ovarian dysmenorrhœa, where the health is becoming seriously impaired, and the case is incurable by other means, to remove the ovaries as a last resort. I must confess that I would be loth to do this, unless the case was an exceptionally bad one, and there was some especial reason why the operation should be performed.

When we consider the effects upon the nutrition and the nervous system of women, of our mode of life, or in other words, of our American civilization, with their badly-balanced and over sensitive nervous organization, and with their predisposition to hysteria and neurotic affections, we must look to improvement of the general health by improved methods of living, by constitutional treatment, by change of location and of surroundings for relief and cure, rather than to surgical procedures on particular organs. I should take the same view regarding oöphorectomy for nymphomania or vaginismus, exhaust other methods of cure first, only resorting to the operation in desperate cases.

Whether the operation will prove of value in cases where epileptic seizures occur habitually at the time of menstruation, is a question requiring farther experiment to determine, but where the attack is clearly dependent upon the nervous disturbance attending the monthly discharge of blood from the uterus, and is not relieved by other means, oöphorectomy is certainly worthy of trial.

Dr. Goodell and Dr. Pallen advise that the ovaries should be removed in all insane women. This is certainly taking very strong ground, and as yet is unwarranted, for we have not had a sufficient number of cases reported where a cure followed the operation to justify such statements.

However, I can understand that in cases of insanity, where an unbalanced mind is manifestly associated with, or caused by a morbid state of the ovaries, that their removal would be justifiable, and would perhaps be the only means left towards effecting a cure. On the other hand, Dr. T. G. Thomas reports three cases of insanity and mania following oöphorectomy, and Dr. Putzel, pathologist of the New York Insane Asylum, found no trace of disease of the ovaries in over one hundred post-mortem examinations made upon the bodies of women dying in that institution. The ovaries may be removed through the vagina by cutting open Douglass' pouch, passing a finger up into the abdominal cavity, drawing the ovary down through the aperture, ligating it, returning the stump, and leaving the incision to heal without sutures, the opening acting as a means of drainage. The objection to this method is, first the difficulty of reaching the ovaries, and next the uncertainty of removing every portion of the diseased gland, owing to adhesions to surrounding structures, consequent upon attacks of pelvic peritonitis or cellulitis. Experience has shown that it is of the first importance that every particle of the ovary be removed, if we wish to get the best results; for if a small portion remains, ovulation still goes on, and the object of the operation will be defeated. If the ovary is prolapsed into Douglass' pouch, and is not fixed by inflammation, and it is desired to remove it, the operation per vaginam is feasible, and not particularly difficult. In all other cases, the removal of the ovaries through an opening in the abdomen has decided advantages. The operation by opening the abdomen is precisely the same as in the operation for ovariotomy; an opening being made in the central line sufficiently large to admit three fingers, or the whole hand if needed, and the condition of the ovaries and fallopian tubes are ascertained. If the operation is performed for uterine hæmorrhage, both ovaries and fallopian tubes are removed; if for other reasons, only that part is removed which is diseased.

The operation is considered more difficult than ovariotomy ordinarily, mainly on account of the rigidity of the abdominal walls, they not having been distended and thinned as in the case of an ovarian tumor, and by reason of the ovaries being small, and sometimes buried in lymph. Hands, sponges, instruments, sutures, and dressings should be absolutely clean if we wish the best results. Listerism should be carried out to the fullest extent, with the exception of the spray, and the after-treatment conducted as after a case of ovariotomy. In his series of cases reported for the removal of the uterine appendages on account of uterine hæmorrhage, Mr. Tait incidentally mentions the fact of his drawing up the retroverted uterus and stitching it to the abdominal wound, and the cases recovered with the uterus retained in its normal position. This, although not mentioned as any special feature, is worthy of notice, as showing what is possible in this direction.

The mortality, so far as I have been able to gather statistics, varies greatly with different operators, and it is very noticeable that the greater the experience of the operator the less the mortality. Speaking of ovariotomy, Dr. Skene, of Brooklyn, a man of learning and skill, says: "This operation differs from all others that I know of, in the number and variety of complications which it affords. It is seldom that two cases exactly alike occur in the practice of any surgeon; hence it is not until a large number of cases have been seen that the operator is prepared to meet all the conditions that may come before him. To the operator of limited practice, the operation in this respect often presents the characteristics of a new investigation."

Mr. Tait says, of his whole number of oöphorectomies, seventy-five in number, the operation being performed for various diseases, the mortality had been eight per cent.; for the last sixty-one cases there have been but three deaths, or a mortality of five per cent.; and the last thirty-five cases of chronic oörphoritis the mortality is but one, or less than three per cent. He says: "It is clearly, therefore, an operation which can be justified by its primary success, only in the hands of a surgeon who has large and constant practice in abdominal surgery, and when it is done by a large number of operators in twos and threes, it can only meet with speedy and well merited condemnation."

Dr. Battey's report at the Medical Congress in London, in 1881, gives the statistics at that time as follows: Of 90 cases of complete removal of the ovaries, 75 per cent. were cured, 17 per cent. were greatly benefited, and 8 per cent. not benefited. Of the incomplete operations, 17 in number, 18 per cent. were cured, 41 per cent. were greatly benefited, and 41 per cent. not benefited.

The mortality of these cases was, in the complete operations, 22 per cent., and in the incomplete operations, 9½ per cent., but it must be remembered that a considerable number of these operations were done before the days of antiseptics, and before abdominal surgery had been perfected as it now is.

It is true that young and inexperienced men do not hesitate to cut open the abdomen for the removal of ovarian tumors, who would hesitate before attempting to perform the operation of removing the lens of the eye for cataract, or performing iridectomy, and yet all must agree that the skill required, and the danger to life involved in the former, is many times greater than in the latter operation.

The secondary effects upon the woman, in case she recovers from the operation, do not differ materially from those incident upon the change of life. The voice, the figure, the dsposition is the same. The only way in which the woman's sexual condition is altered is her inability to bear children; and as this ability is usually destroyed before the operation is undertaken, this argument has but little force.

It has been claimed that it is an operation that is likely to be abused or needlessly performed. This may be true, as it is too often true of many new procedures in surgery; but abuses correct themselves, and I doubt not that the operation is capable of accomplishing much good, and that it will ultimately be recognized as a legitimate procedure in properly selected cases.—*Western Lancet.*

